# Two new cryptic species of *Microhyla* Tschudi, 1838 (Amphibia, Anura, Microhylidae) related to the *M. heymonsi* group from central Vietnam

**DOI:** 10.3897/zookeys.1036.56919

**Published:** 2021-05-05

**Authors:** Chung Van Hoang, Tao Thien Nguyen, Hoa Thi Ninh, Anh Mai Luong, Cuong The Pham, Truong Quang Nguyen, Nikolai L. Orlov, Youhua Chen, Bin Wang, Thomas Ziegler, Jianping Jiang

**Affiliations:** 1 CAS Key Laboratory of Mountain Ecological Restoration and Bioresource Utilization and Ecological Restoration and Biodiversity Conservation Key Laboratory of Sichuan Province, Chengdu Institute of Biology, Chinese Academy of Sciences, Chengdu 610041, China Chengdu Institute of Biology, Chinese Academy of Sciences Chengdu China; 2 University of Chinese Academy of Sciences, Beijing 810000, China University of Chinese Academy of Sciences Beijing China; 3 Vietnam National Museum of Nature, Vietnam Academy of Science and Technology, 18 Hoang Quoc Viet Road, Hanoi, Vietnam Forest Resources and Environment Center Hanoi Vietnam; 4 Institute of Ecology and Biological Resources, Vietnam Academy of Science and Technology, 18 Hoang Quoc Viet Road, Hanoi, Vietnam Vietnam National Museum of Nature, Vietnam Academy of Science and Technology Hanoi Vietnam; 5 Graduate University of Science and Technology, Vietnam Academy of Science and Technology, 18 Hoang Quoc Viet Road, Cau Giay, Hanoi, Vietnam Institute of Ecology and Biological Resources, Vietnam Academy of Science and Technology Hanoi Vietnam; 6 Zoological Institute, Russian Academy of Sciences, Universitetskaya nab 1, St. Petersburg 199034, Russia Graduate University of Science and Technology Hanoi Vietnam; 7 AG Zoologischer Garten Köln, Riehler Strasse 173, D-50735 Cologne, Germany Zoological Institute, Russian Academy of Sciences St. Petersburg Russia; 8 Institute of Zoology, University of Cologne, Zülpicher Strasse 47b, D-50674 Cologne, Germany AG Zoologischer Garten Köln Cologne Germany; 9 Forest Resources and Environment Center, 300 Ngoc Hoi Road, Thanh Tri, Hanoi, Vietnam University of Cologne Cologne Germany

**Keywords:** *
Microhyla
*, new species, central Vietnam, morphology, molecular phylogeny

## Abstract

The *Microhyla heymonsi* species complex from central Vietnam was examined, and based upon morphological and molecular evidence, two new species are described. The discovery of *Microhyla daklakensis***sp. nov.** and *Microhyla ninhthuanensis***sp. nov.** brings the total number of known species in the genus to 46 and the species number of *Microhyla* in Vietnam to 13. The Truong Son Range harbors the highest diversity of the genus *Microhyla* with 11 recorded species so far. However, this apparent micro-endemic diversity is at risk because of habitat loss by deforestation, which highlights the necessity of further research leading to improved conservation measures.

## Introduction

The genus *Microhyla* Tschudi, 1838 currently contains 44 species, which are distributed from India and Sri Lanka eastwards to the Ryukyu Archipelago of Japan and southwards to Indonesia (Hoang et al. 2020; Gorin et al. 2021; Frost 2021). Recently, Gorin et al. (2021) described a new genus *Nanohyla* from the *Microhyla* – *Glyphoglossus* assemblage (currently called the *Microhyla* – *Nanohyla* – *Glyphoglossus* assemblage). Recent studies have discovered highly divergent mitochondrial DNA lineages indicating a high degree of undiagnosed diversity in the genus (Hasan et al. 2012, 2014, 2015; Howlader et al. 2015; Matsui et al. 2005, 2011, 2013; Seshadri et al. 2016; Wijayathilaka et al. 2016; Yuan et al. 2016; Zhang et al. 2018; Nguyen et al. 2019; Li et al. 2019; Poyarkov et al. 2019; Hoang et al. 2020). Remarkably, 23 new species have been described during the last decade (Frost 2021).

**Figure 1.**
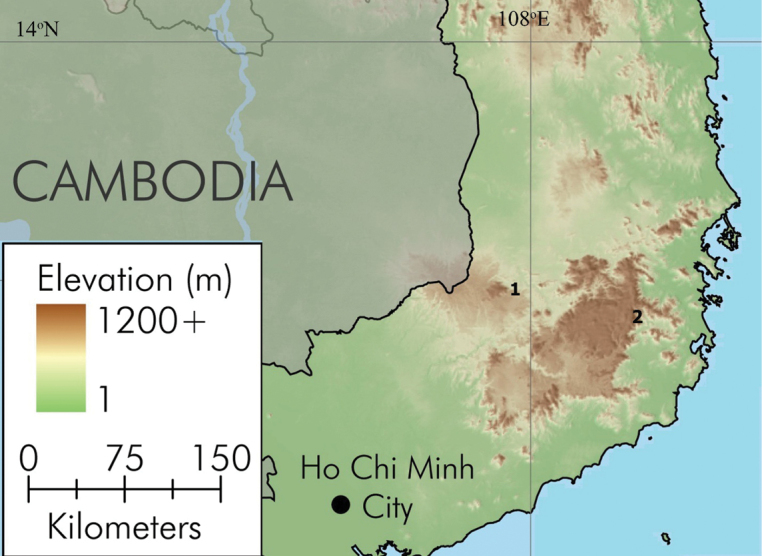
Map showing the type localities of *Microhyla ninhthuanensis* sp. nov. in Ninh Thuan Province (**2**) and *Microhyla daklakensis* sp. nov. in Dak Lak Province (**1**) in Tay Nguyen Plateau (Central Highlands) of Vietnam (EJ Sterling and K Koy kindly provided the map).

In Vietnam, nine species of *Microhyla* have been recorded to date (Frost 2021), with the greatest species diversity occurring in the central and southern parts of the Truong Son Range, also known as the Central Highlands or the Tay Nguyen Plateau (Fig. 1). This mountain range harbors the highest diversity of amphibians in the Indochina region with a high degree of local endemism, and it is considered as a hotspot for new species discoveries (Bain and Hurley 2011; Geissler et al. 2015; Nguyen et al. 2019). This mountain range also appears to be one of the centers of radiation for the genus *Microhyla* (Poyarkov et al. 2014; Gorin et al. 2020). The following species have been described since 2010: *M. aurantiventris* Nguyen, Poyarkov, Nguyen, Nguyen, Tran, Gorin, Murphy & Nguyen, 2019; *M. darevskii* Poyarkov, Vassilieva, Orlov, Galoyan, Tran, Le, Kretova & Geissler, 2014; *M. mukhlesuri* Hasan, Islam, Kuramoto, Kurabayashi & Sumida, 2014; *M. neglecta*﻿ Poyarkov, Nguyen, Trofimets & Gorin, 2020; *M. pineticola* Poyarkov, Vassilieva, Orlov, Galoyan, Tran, Le, Kretova & Geissler, 2014 (Bain and Nguyen 2004; Hoang et al. 2013; Inger et al. 1999; Poyarkov et al. 2014; Nguyen et al. 2019; Poyarkov et al. 2020).

Heymon’s narrow-mouthed frog, *Microhyla heymonsi* Vogt, 1911, was originally described from Taiwan based on eight male specimens (ZMB 54906–54913). Apart from Taiwan, this species has a wide distribution (Jang-Liaw and Chou 2015), and is reported from across East and Southeast Asia, from mainland China through Sumatra (Amphibia Web 2020; Frost 2021). Recently, Garg et al. (2019) demonstrated that *M. heymonsi* represents a complex of species, and that the current number of recognized *Microhyla* species may be underestimated. Garg et al. (2019) revealed six and Gorin et al. (2020) revealed seven or eight genetic lineages within *M.* ‘*heymonsi*’.

During our recent field surveys in Kon Tum, Dak Lak, Lam Dong, and Ninh Thuan provinces in central Vietnam between 2016 and 2019, a number of microhylid frog specimens were collected that morphologically resembled *M.* ‘*heymonsi*’. However, morphological and molecular analyses showed that these populations represent independent evolutionary lineages. The population from Lam Dong Province was recently described as *M. neglecta* (Poyarkov et al. 2020). Two further populations remained that could not be assigned to any known species of *Microhyla*. Herein, we describe these two populations of *Microhyla* from central Vietnam as two new species.

## Materials and methods

### Sampling

Field surveys were conducted in Kon Tum, Dak Lak, Lam Dong, and Ninh Thuan provinces, Vietnam (Fig. 1) in June 2016, April and May 2018 and April 2019 by C.V. Hoang, A.M. Luong, Y.T. Nguyen, H.T. Ninh, N. L. Orlov, L. Iogansen (hereafter C.V. Hoang et al.). Geographic coordinates and elevations were obtained using a Garmin GPSMAP 78S (WGS 84 data). After photographing specimens in life, they were euthanized in a closed vessel with a piece of cotton wool containing ethyl acetate (Simmons 2002). Specimens were fixed in 70% ethanol for five hours, and then later transferred to 70% ethanol for permanent storage. Tissue samples were preserved separately in 70% ethanol prior to specimen fixation. Specimens referred to in this paper are deposited in the collections of the Vietnam National Museum of Nature (**VNMN**), Hanoi, Vietnam; Institute of Ecology and Biological Resources (**IEBR**), Hanoi, Vietnam; Chengdu Institute of Biology (**CIB**), Chengdu, Sichuan, China; and the Zoological Institute of the Russian Academy of Sciences (**ZISP**), St. Petersburg, Russia. Sex was determined by the direct observation of calling males in life or by gonadal dissection. Further information on the specimens is provided in Suppl. material 1: Table S1.

### Molecular analyses

Extraction of genomic DNA from 41 tissue samples (Suppl. material 1: Table S2) was carried out using the TIANamp Genomic DNA kit (Tiangen Biotech, Beijing, China), following the manufacturers’ instructions. We amplified a 1979 base pair (bp) fragment that encodes part of the 12S rRNA gene, the complete tRNA Val gene, and part of the 16S rRNA gene that was used recently for *Microhyla* (Nguyen et al. 2019). The polymerase chain reaction (PCR) was performed using an Eppendorf PCR machine in 25 μl reactions containing 12 μl of Mastermix, 6 μl of water, 1 μl of each primer at a concentration of 10 pmol/μl, and 5 μl of DNA. We have amplified in multiple fragments: Fragment first, primers: 12SAL (5’-AAACTGGGATTAGATACCCCACTAT-3’; forward), 16S2000H (5’-GTGATTAYGCTACCTTTGCACGGT-3’; reverse) (Zhang et al. 2008) used in the PCR and sequencing; and fragment second, primers: LR-N-13398 (5’-CGCCTGTTTACCAAAAACAT -3’; forward), LR-J 12887 (5’-CCGGTCTGAACTCAGATCACGT -3’; reverse) (Simon 1994) used in the PCR and sequencing. PCR conditions: 94 °C for 5 minutes of initial denaturation; with 35 cycles of denaturation at 94 °C for 30 s, annealing at 56 °C for 30 s, and extension at 72 °C for 45 s; and the final extension at 72 °C for 7 minutes. PCR products were sent to Tsingke Biological Technology company for sequencing (http://www.tsingke.net). The obtained sequences were deposited in GenBank under the accession numbers MT808928–MT808963 and MT819964–MT819968 (Suppl. material 1: Table S2).

In addition to the 41 sequences of the collected samples in this work, we used 76 available sequences of 12S rRNA–16S rRNA from GenBank (Garg et al. 2019) for phylogenetic analyses. Sequences of *Kaloula pulchra* Gray, 1831 were included in the analysis as outgroup (Van Bocxlaer et al. 2006). Locality information and accession numbers for all sequences included in the analysis can be found in Suppl. material 1: Table S2.

Phylogenetic trees were constructed by using Maximum Likelihood (ML) and Bayesian Inference (BI) analyses. Chromas Pro software (Technelysium Pty Ltd., Tewantin, Australia) was used to edit the sequences, and then aligned using the ClustalW (Thompson et al. 1997) option in MEGA 7.0 (Kumar et al. 2016) with default parameters and subsequently optimized manually in BioEdit 7.0.5.2 (Hall 1999). We then checked the initial alignments by eye and made slight adjustments. Prior to Bayesian tests, phylogenetic analyses were performed in MrBayes 3.2 (Ronquist et al. 2012). We chose the optimum substitution models for entire sequences using Kakusan 4 (Tanabe 2011) based on the Akaike information criterion (AIC). The best model selected for ML was the general time reversible model (GTR: Tavaré 1986) with a gamma shape parameter (G: 0.202 in ML and 0.226 in BI). The BI summarized two independent runs of four Markov Chains for 10 000 000 generations. A tree was sampled every 100 generations and a consensus topology was calculated for 70 000 trees after discarding the first 30 001 trees (burn-in = 3 000 000) (Nguyen et al. 2017). We checked parameter estimations and convergence using Tracer version 1.5 (Rambaut and Drummond 2009). The strength of nodal support in the ML tree was analyzed using non-parametric bootstrapping (MLBS) with 1000 replicates. We regarded tree nodes in the ML tree with bootstrap values of 75% or greater as sufficiently resolved (Hillis and Bull 1993; Huelsenbeck and Hillis 1993), and nodes with a BPP of 95% or greater as significant in the BI analysis (Leaché and Reeder 2002).

### Morphological analysis

All measurements were taken from 63 preserved specimens (Suppl. material 1: Table S4) with a digital caliper to the nearest 0.01 mm under a dissecting microscope. The following morphological characteristics were taken following Matsui (2011); Matsui et al. (2013), and Poyarkov et al. (2014), with some modifications:

**SVL** snout-vent length (measured from the tip of snout to cloaca);

**HL** head length (measured from tip of snout to hind border of jaw angle, but not measured parallel with the median line as done by Matsui 2011);

**SL** snout length (measured from the anterior corner of eye to the tip of snout);

**EL** eye length (measured as the distance between the anterior and posterior corners of the eye);

**N-EL** nostril-eye length (measured as the distance between the anterior corner of the eye and the nostril);

**HW** head width (measured as the maximum width of the head on the level of mouth angles in ventral view);

**IND** internarial distance (measured as the distance between central points of nostrils);

**IOD** interorbital distance (measured as the shortest distance between the medial edges of eyeballs in dorsal view);

**UEW** upper eyelid width (measured as the widest distance from the medial edge of eyeball to the lateral edge of the upper eyelid);

**FLL** forelimb length (measured as length of straightened forelimb to tip of third finger);

**LAL** lower arm and hand length (measured as distance from elbow to tip of third finger);

**HAL** hand length (measured from proximal end of outer palmar [metacarpal] tubercle to tip of third finger);

**IPTL** inner palmar tubercle length (measured as maximal distance from proximal to distal ends of inner palmar tubercle);

**OPTL** outer palmar tubercle length (measured as maximal diameter of outer palmar tubercle);

**HLL** hindlimb length (measured as length of straightened hindlimb from groin to tip of fourth toe);

**TL** tibia length (taken as the distance between the knee and tibiotarsal articulation);

**FL** foot length (measured from distal end of tibia to tip of toe IV);

**IMTL** inner metatarsal tubercle length (taken as maximal length of inner metatarsal tubercle);

**1TOEL** first toe length (from distal end of inner metatarsal tubercle to tip of first toe);

**OMTL** outer metatarsal tubercle length;

**1FW** first finger width (measured at the distal phalanx);

**1–3FLO** finger lengths, outer side (**O**) of the first-third;

**2–4FLI** finger lengths, inner side (**I**) of the second-fourth;

**2–4FDW** finger disk diameters;

**1–5TDW** toe disk diameters.

Terminology for describing eye coloration in life followed Glaw and Vences (1997) and webbing formula followed Savage (1975).

### Morphological comparisons

A total of 45 species (Suppl. material 1: Table S5) was compared based on examined specimens (Suppl. material 1: Table S4) and from data in the literature: Boulenger (1897, 1900, 1920); Smith (1923); Parker (1928, 1934); Andersson (1942); Bourret (1942); Parker and Osman (1948); Pillai (1977); Inger and Frogner (1979); Inger (1989); Dutta and Ray (2000); Bain and Nguyen (2004); Das et al. (2007); Das and Haas (2010); Fei et al. (2012); Matsui (2011); Matsui et al. (2013); Hasan et al. (2014); Poyarkov et al. (2014); Howlader et al. (2015); Seshadri et al. (2016); Wijayathilaka et al. (2016); Khatiwada et al. (2017); Zhang et al. (2018); Nguyen et al. (2019); Garg et al. (2019); Poyarkov et al. (2019); Li et al. (2019); and Hoang et al. (2020).

### Principal component analysis (PCA)

Measurements were used to compare the morphometric difference between the four males and six females of the population from Dak Lak Province and the nine males and two females of the population from Ninh Thuan Province vs. the eight males and two females of *M.* ‘*heymonsi*’ from Kon Tum Province. All statistical analyses were performed using PAST 2.17b software (Hammer et al. 2001).

## Results

### Sequence variations

The final alignment of 12S rRNA–16S rRNA contained 1979 numbers of characters. Of these, 1222 sites were conserved and 731 sites exhibited variation, with 587 characters being parsimony-informative. The transition-transversion bias (R) was estimated as 1.279. Nucleotide frequencies were A = 31.64%, T = 23.96%, C = 23.09%, and G = 21.31% (data for ingroup only).

### Interspecific uncorrected p-distances

The genetic divergence of the population from Ninh Thuan Province and its congeners ranged from 3.6% (*M. heymonsi* sensu stricto from Taiwan) to 13.2–13.4% (*M. laterite*). The genetic divergence of the population from Dak Lak Province and its congeners ranged from 2.4–2.9% (the *M.* ‘*heymonsi*’ population from Kon Tum Province) to 12.4–13.4% (*M. nanapollexa*). These values were higher than the genetic distances between other recognized species of *Microhyla* (i.e., 2.4% between *M. fanjingshanensis* and *M. beilunensis*, *M. borneensis* and *M. malang*; 2.4% between *M. okinavensis* and *M. beilunensis*; 2.2% between *M. okinavensis* and *M. mixtura*) (Suppl. material 1: Table S3). Our results support those of Garg et al. (2019) and Gorin et al. (2020), clearly indicating that the *M.* ‘*heymonsi*’ clade represents a complex of several species, either representing previously available names or so far unrecognized diversity.

### Phylogenetic relationships

**Figure 2.**
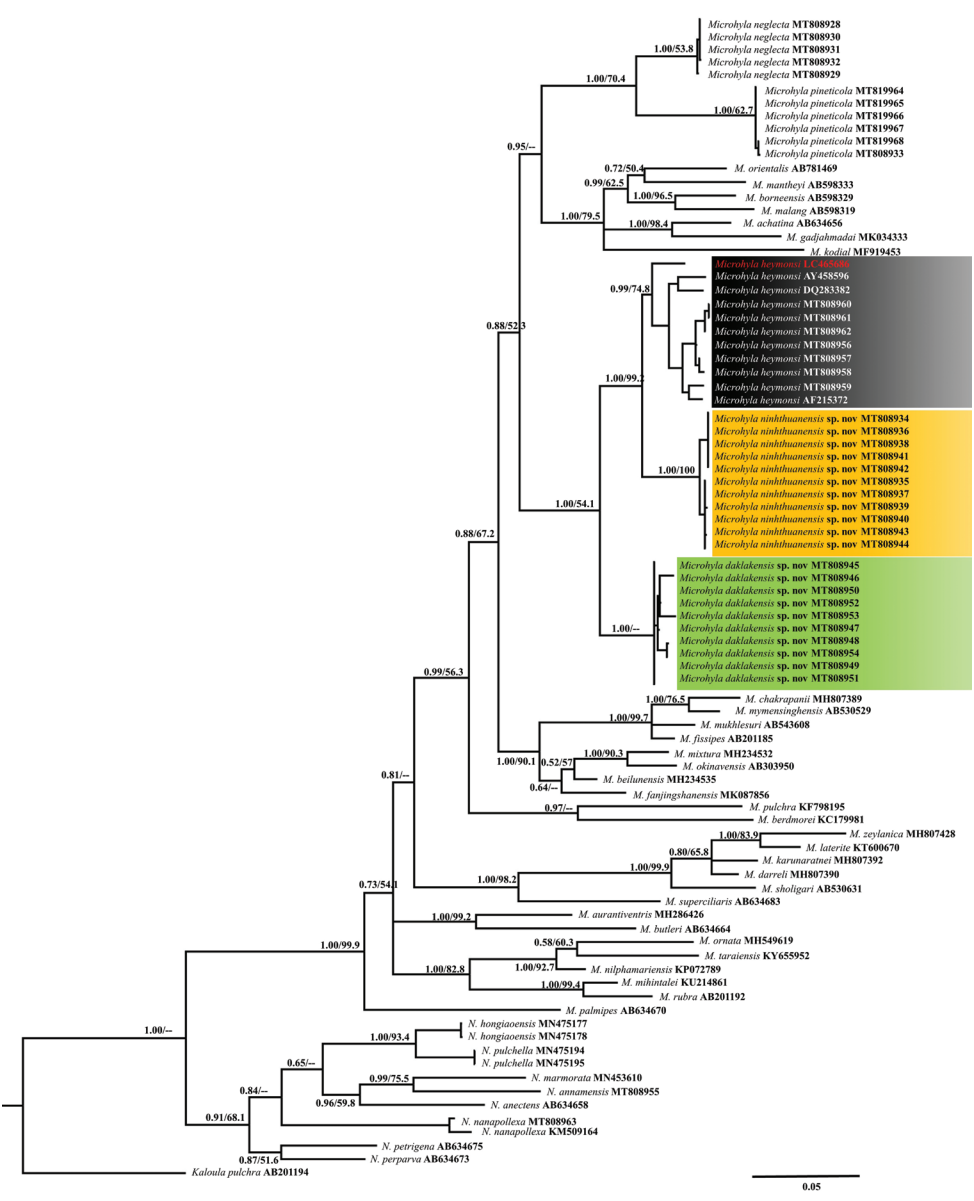
Bayesian Inference of the matrilineal genealogy of *Microhyla* derived from the analysis of 1979 bp of 12S rRNA–16S rRNA mtDNA sequences. Numbers above and below branches are Bayesian posterior probabilities and ML bootstrap values. The scale bar represents 0.05 nucleotide substitutions per site.

The BI and ML analyses produced topologies with –ln L = 15327.579 and 12672.916, respectively. BI and ML analyses obtained similar topologies (Fig. 2) that differed only at several poorly supported basal nodes. Our matrilineal genealogy was consistent with previous studies based on mt DNA (e.g., Matsui et al. 2005, 2011; Hasan et al. 2012; Matsui et al. 2013; Howlader et al. 2015; Wijayathilaka et al. 2016; Seshadri et al. 2016; Yuan et al. 2016; Khatiwada et al. 2017; Nguyen et al. 2019; Garg et al. 2019; and Hoang et al. 2020). As shown in previous molecular analyses (Matsui et al. 2011; Peloso et al. 2016), Asian *Microhyla* species were divided into two geographical subgroups: the Southeast Asian subgroup was recently named as *Nanohyla* by Gorin et al. (2021), with eight known species: *N. annamensis*, *N. annectens*, *N. hongiaoensis*, *N. marmorata*, *N. nanapollexa*, *N. perparva*, *N. petrigena*, and *N. pulchella*; and Pan-Asian, including all other South, Southeast, and East Asian members (Fig. 2). Among the known taxa, molecular data are presented for the first time for four poorly known species: *N. annamensis*, *N. hongiaoensis*, *N. pulchella*, and *M. pineticola*. Molecular results further revealed *M. heymonsi* to represent a complex consisting of multiple species.

### Morphological analysis

The two new forms could be assigned to the genus *Microhyla* based both on the molecular phylogenetic data and the following morphological characters: size relatively small; maxillary and vomerine teeth absent; vomer divided into two parts, disappearing at the posterior edge of the choana; tongue round posteriorly; skin smooth or with tubercles; tympanum hidden; palate with one or two rows of horizontal skin ridges; fingers without webbing; toes slightly webbed or free of webbing; metacarpal tubercles two or three; and the absence of skin ridge or skin projection between the subarticular tubercles of toes III and IV.

The two new forms differ from other known species of *Microhyla* by having a medium body size, stocky habit, round snout, smooth skin on dorsum, disks on distal end of toes present, dorsal median longitudinal grooves on finger disks present, superciliary tubercles absent, light dorsomedial vertebral line present (morphological characters and distribution data for each species are summarized in Suppl. material 1: Table S5).

The two new forms of *Microhyla* from Dak Lak and Ninh Thuan provinces can also be separated from *M.* ‘*heymonsi*’ based on morphometric data. We extracted three principal component axes in the PCA result with eigenvalues greater than 0.01, the first two component axes accounted for 93.49% (in males) and 81.80% (in females) of the variation (Suppl. material 1: Table S6). The first two principal component axes could separate the new forms from *M.* ‘*heymonsi*’ by 23 characters (Fig. 7), mainly based on limb and head measurements, namely: SVL, HL, HW, SL, EL, N-EL, IND, IOD, UEW, FLL, LAL, HAL, IPTL, OPTL, 1FL, 3FDW, HLL, TL, FL, 1TOEL, IMTL, OMTL, and 3TDD (Suppl. material 1: Table S6). In males, species with a larger and positive score on PC1 reflected shorter SVL, HL, HW, SL, EL, N-EL, IND, IOD, UEW, FLL, LAL, HAL, IPTL, OPTL, 1FL, HLL, TL, and FL; while a negative score signified smaller 3FDW, 1TOEL, IMTL, OMTL, and 3TDD. The PC2 with positive scores were associated with species with larger morphological traits with all 23 characters, whereas no negative scores were associated with species (Suppl. material 1: Table S6). In females, species with a larger and positive score on PC1 reflected shorter SVL, HL, HW, SL, EL, N-EL, IOD, UEW, FLL, LAL, HAL, IPTL, OPTL, 1FL, HLL, TL, and FL; while a negative score signified smaller IND, 3FDW, 1TOEL, IMTL, OMTL, and 3TDD. The PC2 with positive scores were associated with species with larger SVL, HL, HW, SL, EL, N-EL, IOD, UEW, FLL, LAL, HAL, IPTL, OPTL, 1FL, HLL, TL, FL, 1TOEL, IMTL, OMTL, and 3TDD while a negative score signified smaller IND and 3FDW (Suppl. material 1: Table S6).

### Taxonomic conclusions

Based upon the phylogenetic analyses of 12S rRNA–16S rRNA sequences, the two populations clearly differ from all other species of *Microhyla* for which comparable genetic data are available, and the observed differences in mtDNA sequences were congruent with other morphological data. Accordingly, we describe the two new species as follows.

#### Microhyla ninhthuanensis sp. nov.

Taxon classificationAnimaliaAnuraMicrohylidae

F53571D7-7DA7-5381-AAFD-316B57698633

http://zoobank.org/C458EC2A-C0AE-4BF6-B886-772A85A81911

**Figure 3.**
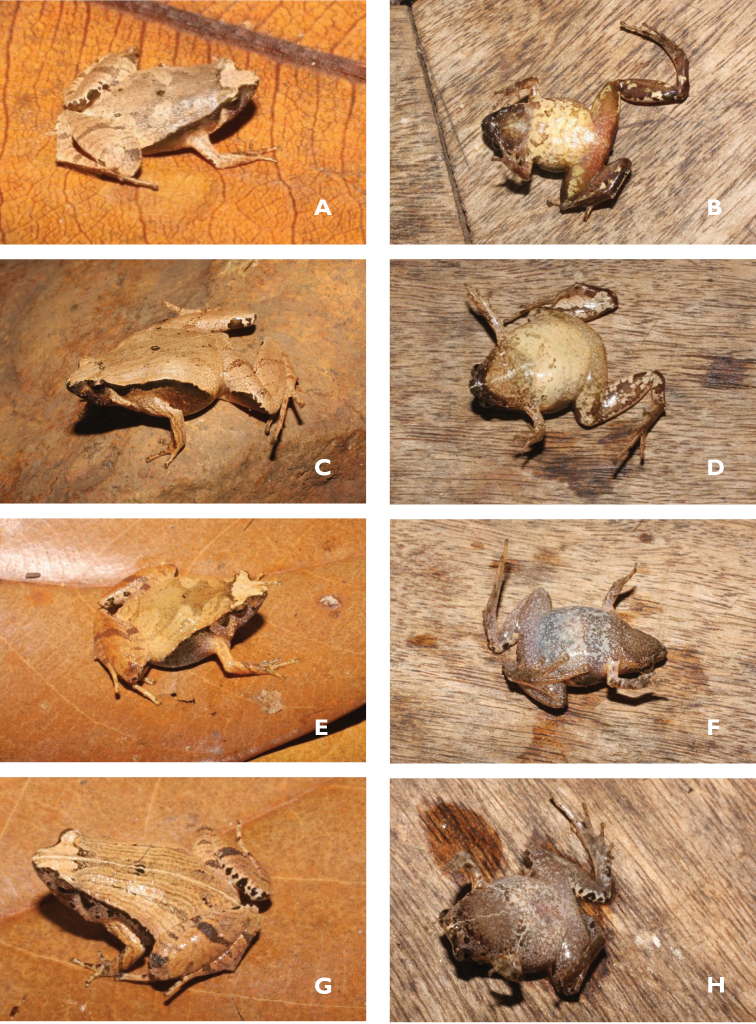
Dorsolateral and ventral views of the specimens in life: comparative specimen of *Microhyla neglecta* (VNMN 07344, male) (**A, B**); (VNMN 07673, female) (**C, D**); comparative specimen of *M. pineticola* (VNMN 07441, female) (**E, F**) and (VNMN 07455, male) (**G, H**). Photographs by CV Hoang and NL Orlov. Dorsolateral and ventral views of the specimens in life: comparative specimen of *M. ‘heymonsi*’ (KPMĐ2018.42, male) (**I, J**); the holotype of *Microhyla daklakensis* sp. nov. (VNMN 06877, male) (**K, L**); the paratype of *Microhyla ninhthuanensis* sp. nov. (ZISP 14254 (HAO186), female) (**M**). Photographs by CV Hoang and NL Orlov.

**Figure 4.**
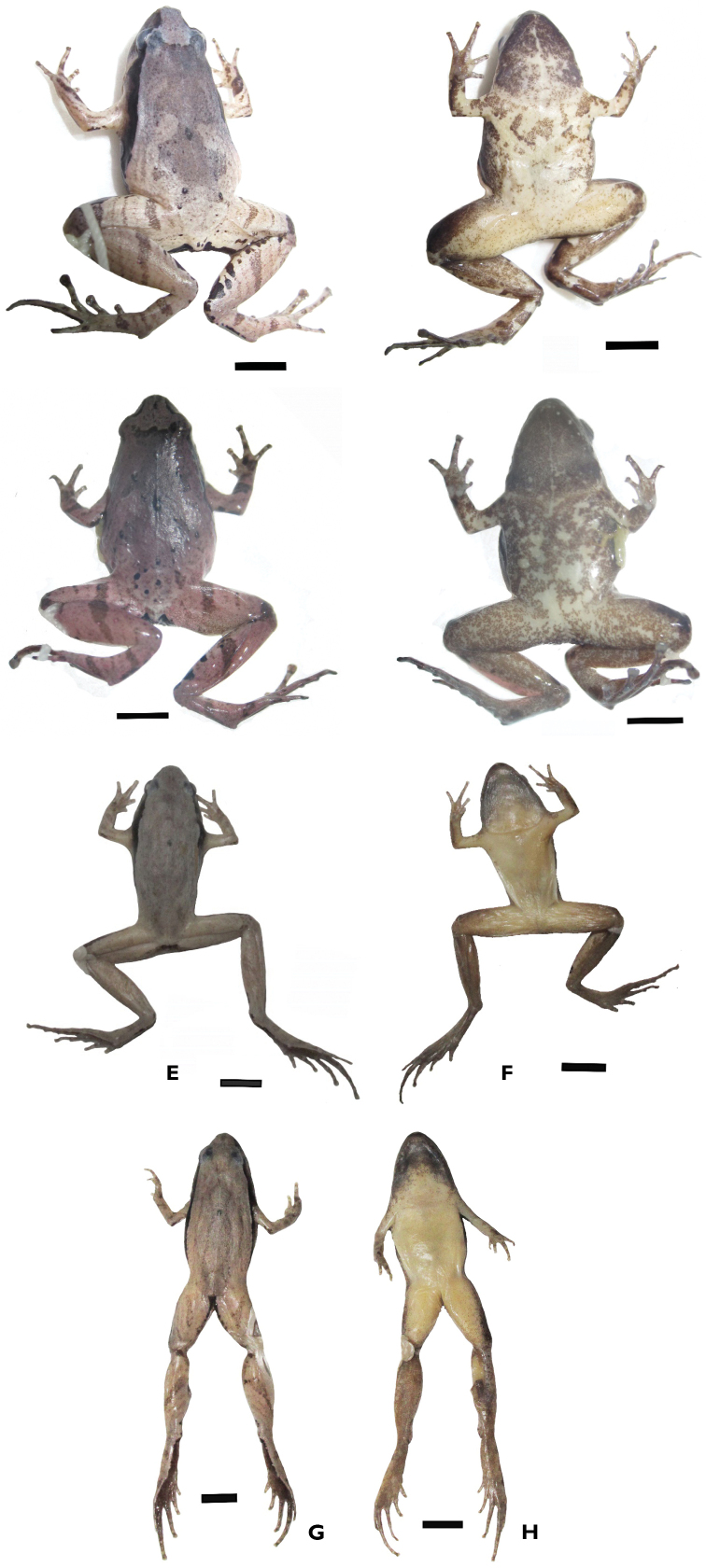
Dorsolateral and ventral views of the specimens in preservative: comparative specimen of *Microhyla neglecta* (VNMN 07344, male) (**A, B**), comparative specimen of *M. pineticola* (VNMN 07719, male) (**C, D**). Photographs by CV Hoang. Scale bars 4 mm. Dorsolateral and ventral views of the specimens in preservative: the holotype of *Microhyla daklakensis* sp. nov. (VNMN 06877, male) (**E, F**); and the holotype of *Microhyla ninhthuanensis* sp. nov. (VNMN 2021.01, male) (**G, H**). Photographs by CV Hoang. Scale bars 4 mm.

**Figure 5.**
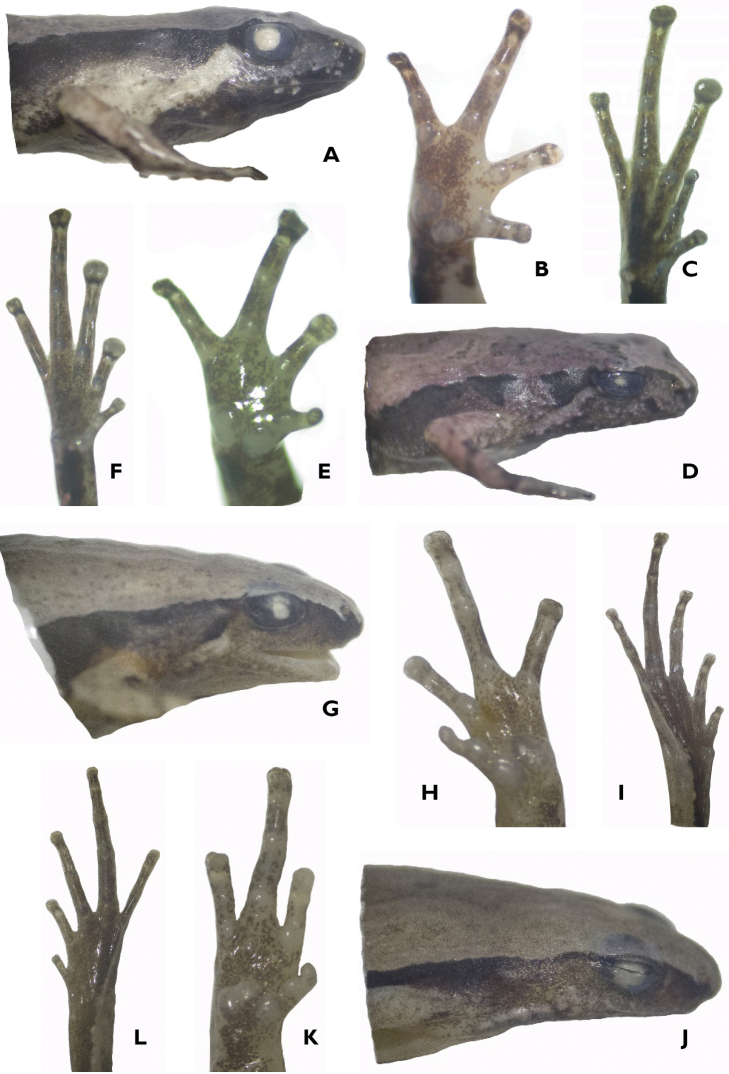
Lateral view of the head, right hand and right food of *Microhyla neglecta* (VNMN 07344, male) (**A, B, C**); comparative specimen of *Microhyla pineticola* (VNMN 07719, male) (**D, E, F**); *Microhyla daklakensis* sp. nov. (VNMN 06877, male) (**G, H, I**); and *Microhyla ninhthuanensis* sp. nov. (HAO73, male) (**J, K, L**). Photographs by CV Hoang.

Figures 3M , 4G, H , 5J, K, L ; Table 1 ; Suppl. material 1: Tables S4, S5

##### Holotype.

VNMN 2021.01 (HAO 73), adult male, collected in Phuoc Binh National Park, Bac Ai District, Ninh Thuan Province, Vietnam (11°59'3.71"N, 108°44'51.63"E, ca. 305 m a.s.l., Fig. 1); leg. C.V. Hoang et al., 28 April 2018.

##### Paratypes.

(n = 10) All collected by C.V. Hoang et al. at the same location as the holotype: IEBR.A 4841–4843 (HAO74, HAO76, HAO77), VNMN 2021.02–2021.05 (HAO78, HAO79, HAO80, HAO184), CIB (HAO185) eight adult males and ZISP 14253–14254 (HAO75, HAO186) two adult females, collected on Phuoc Binh National Park, Bac Ai District, Ninh Thuan Province, Vietnam (11°59'3.71"N, 108°44'51.63"E, ca. 305 m a.s.l., Fig. 1), 28 April 2018.

**Table 1.** Selected diagnostic characters for the comparisons between the species of the *Microhyla heymonsi* group.

	*M.* ‘*heymonsi*’	*Microhyla daklakensis* sp. nov.	*Microhyla ninhthuanensis* sp. nov.
SVL M	16.5–22.0	17.7–20.1	17.3–18.8
SVL F	18.0–26.5	22.9–26.8	21.6–23.6
Habit	Stocky	Stocky	Stocky
Snout profile	rounded, obtusely pointed	rounded	rounded
Dorsal skin	smooth	smooth	smooth
F1 vs. F2	F1 ≤ ½ F2	F1 > ½ F2	F1 ≤ ½ F2
Disks on distal end of fingers	present	present	present
Dorsal median longitudinal line grooves on finger disks	usually present	present	present
Disks on distal end of toes	present	present	present
Dorsal peripheral grooves on toe disks	usually present	present	present
Presence or absence of superciliary tubercles	absent	absent	absent
Presence or absence of light dorsomedial (vertebral) line	present	present	present
Tibiotarsal articulation	shorter than snout	shorter than snout	shorter than snout
Foot webbing	I2 – 2½II2 – 3III3 – 4IV4⅓ – 3V	I2 – 2½II2 – 3III3 – 4IV4⅓ – 3V	I2 – 2½II2 – 3III3 – 4IV4⅓ – 3V
Distribution	S China, NE India, SE Asia to Sumatra	Dak Lak	Ninh Thuan

##### Diagnosis.

*Microhyla ninhthuanensis* sp. nov. is distinguished from its congeners by a combination of the following morphological characters: 1) body stocky, size medium (SVL 17.3–18.8 mm, n = 9 males; 21.6–23.6 mm, n = 2 females). 2) dorsum smooth; 3) head triangular, snout round in profile; 4) finger I shorter than one-half the length of finger II; 5) tips of all outer fingers dilated, forming disks, with a median longitudinal groove visible dorsally; 6) tips of all toes distinctly dilated into disks, with a weak median longitudinal groove visible dorsally, producing the appearance of two scutes; 7) inner metacarpal tubercle oval and prominent, paired outer metacarpal tubercle divided by a waist into two equal-sized parts: outer part quite round, inner part quite crescent; 8) tibiotarsal articulation of straightened limb not reaching snout; 9) webbing basal: I2 – 2½II2 – 3III3 – 4IV4⅓ – 3V; 10) inner metatarsal tubercles oval, prominent and outer metatarsal tubercles round; 11) upper eyelid without supraciliary spines; 12) narrow faint brown stripe extending from rear corner of eye to axilla; 13) light thin vertebral stripe present, canthus rostralis with dark lines; 14) small dark round spot at mid-dorsum, divided by a light vertebral stripe; 15) dorsum pinkish brown with dark brown marking in X-shape between eyes and arm, along vertebral and dorsolateral region stripes form wavy dust strip towards the groin, a small dark marking ‘ ()’-shaped in the center of the dorsum and mid-dorsal line; 16) an even black lateral stripe from above arm, almost reaching groin; 17) chin dark grey; throat white with scattered dark grey dusting; chest and belly creamy white.

##### Description of holotype.

Habitus stocky, size medium, SVL 18.20 mm; head wider than long (HL/HW 0.83); snout long, abruptly round in dorsal view, projecting beyond margin of lower jaw, longer than diameter of eye (SL/EL 1.24); eyes small, slightly protuberant, pupil round (Fig. 4G); dorsal surface of head flat, loreal region acute; indistinct canthus rostralis; nostril oval, lateral, closer to tip of snout (N-EL 1.33) than to eye (EL 1.95); interorbital distance wide, greater than internarial distance (IOD/IND 1.28); and internarial distance wide, greater than upper eyelid width (IND/UEW 1.18); tympanum hidden, supratympanic fold weak, from posterior corner of eye to arm insertion; vomerine teeth absent, tongue without papillae, oval and free at the rear half of its length; slit-like openings to a small vocal sac.

Forelimbs short, about three times shorter than hindlimbs (FLL/HLL 0.31); hand two times shorter than forelimb length (HAL/FLL 0.43); fingers slender, free of webbing, round in cross-section, skin fringes of fingers weak; first finger shorter than one-half the length of the second finger (1FLO/2FLO 0.44), second finger slightly shorter than fourth (2FLI/4FLI 0.98), latter much longer than first, and much shorter than third (2FLI/3FLI 0.74); relative finger lengths: I < II < IV < III (Fig. 5K). All disks bearing narrow peripheral grooves, dorsal finger tips with median longitudinal grooves producing the appearance of two scutes; relative finger disk widths: I < IV < II < III; nuptial pad absent; subarticular tubercles on fingers distinct, round, finger subarticular tubercle formula: 1:1:2:2 (given for fingers I:II:III:IV, respectively); inner metacarpal tubercle (IPTL 0.48) oval, prominent; a paired outer metacarpal tubercle divided by a waistline into two equal-sized parts: outer part quite round, inner part quite crescent (OPTL 0.47) (Fig. 5K).

Hindlimbs slender and slightly short (HLL 31.62), tibia length longer than half of snout-vent length (TL/SVL 0.58); tibiotarsal articulation at straightened limb not reaching snout; foot longer than tibia (FL/TL 1.32); relative toe lengths: I<II<V<III<IV; tarsus smooth, inner tarsal fold absent; tips of all toes distinctly dilated into disks, slightly wider than those of fingers (3TDW 0.53, 3FDW/3TDW 0.93), dorsally all toes with median longitudinal grooves at disks; relative toe disk widths: I<V<II<III=IV; webbing between toes basal and poorly developed, webbing formula: I2 – 2½II2 – 3III3 – 4IV4⅓ – 3V; subarticular tubercles prominent, all present, circular, formula 1, 1, 2, 3, 2; inner metatarsal tubercle elongated, oval, large and prominent, length (IMTL 0.78); outer metatarsal tubercle round, elevated and very well distinct, smaller (OMTL 0.38) than length of inner metatarsal tubercle (Fig. 5L).

Dorsal surface of head and body smooth, flank shagreened, dorsal surface smooth, including fingers and toes, fore and hind limbs; ventral surfaces smooth.

##### Coloration of holotype in life.

Dorsal surface of head and trunk pinkish brown with a dark brown marking in X-shape between eyes to arm, dorsolateral stripes form wavy dust strip towards the groin; a small dark marking in ‘ ()’-shape in the center of the dorsum and a mid-dorsal line extending from the tip of snout to vent. Flanks and lateral surface of head dark, a dark lateral stripe running from snout tip to nostril, fading towards upper jaw. Chin dark grey; throat white with scattered dark grey dusting; chest and belly creamy white. Limbs dorsally with narrow indistinct dark brown cross-bars; fingers and toes dorsally brown with dark brown cross-bars; forelimbs ventrally creamy white, hindlimbs ventrally with creamy white thigh becoming dark grey toward shank, foot. Iris bicolored, golden in upper one-third, dark copper in its lower two-thirds; pupil oval, horizontal, black.

##### Coloration of holotype in preservative.

After preservation in ethanol, dorsal coloration changed from light brown to greyish pink (Fig. 4G), ventral surface of chest, belly, and limbs changed from creamy white to whitish beige (Fig. 4H); dorsal pattern: dark spots on dorsum and stripes on dorsal surfaces of limbs unchanged, dark brown pattern changed to dark grey; iris completely black, pupil round, white.

##### Variation.

Specimens vary in body size, dorsal markings, and black scapular spots. Adult males smaller than adult females, adult males with small vocal sac (Suppl. material 1: Table S4).

##### Comparisons.

*Microhyla ninhthuanensis* sp. nov. is morphologically most similar to *M. heymonsi* sensu stricto (Fig. 2), but differs by having: 1) a snout round in profile (vs. snout obtusely pointed in *M. heymonsi* sensu stricto), 2) pinkish brown dorsal surface with a dark brown marking in X-shape between eyes to insertion of the arms (vs. dorsal surface red to grayish red with a dark brown marking in X-shape and a vague V-shape dark brown marking in *M. heymonsi* sensu stricto (Vogt, 1913). Detailed comparisons between *M. ninhthuanensis* sp. nov. and other members of the *M. heymonsi* group are shown in Table 1 and Suppl. material 1: Table S5.

##### Etymology.

Specific epithet is in reference to the type locality, Ninh Thuan Province. We recommend “Ninh Thuan narrow-mouth frog” as the common English name and “Nhái bầu ninh thuận” as the Vietnamese name.

##### Natural history.

**Figure 6.**
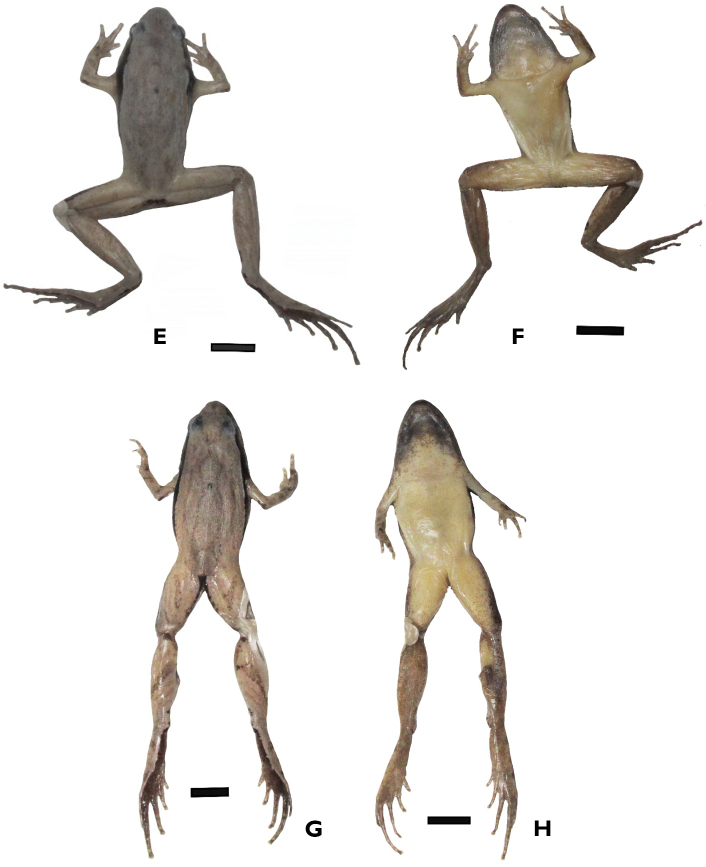
Habitat of *Microhyla daklakensis* sp. nov. in Nam Ka NR, Dak Lak Province (**A**); and of *Microhyla ninhthuanensis* sp. nov. in Phuoc Binh NP, Ninh Thuan Province (**B**), Vietnam. Photos by C. V. Hoang.

All specimens were collected at night from 19:00 to 23:00 h on the ground near the banks of a small stream in the forest and on the sides of a recently constructed road next to the devastated forests (Fig. 6B). Larval stages and eggs of the new species are unknown.

##### Distribution.

*Microhyla ninhthuanensis* sp. nov. is currently only known from the type locality in Phuoc Binh National Park, Ninh Thuan Province, Vietnam (Fig. 1). The species was recorded at an elevation of ca. 300 m a.s.l.

##### Conservation status.

Currently, the evergreen forest in Phuoc Binh National Park is connected with other forests in Tay Nguyen Plateau. Based on its habitat and altitudinal range, the new species is likely to be endemic to Tay Nguyen Plateau. However, the extent of its actual distribution range requires further study. Given the available information, we suggest *Microhyla ninhthuanensis* sp. nov. be considered as Data Deficient following IUCN’s Red List categories (IUCN Standards and Petitions Subcommittee 2001).

**Figure 7.**
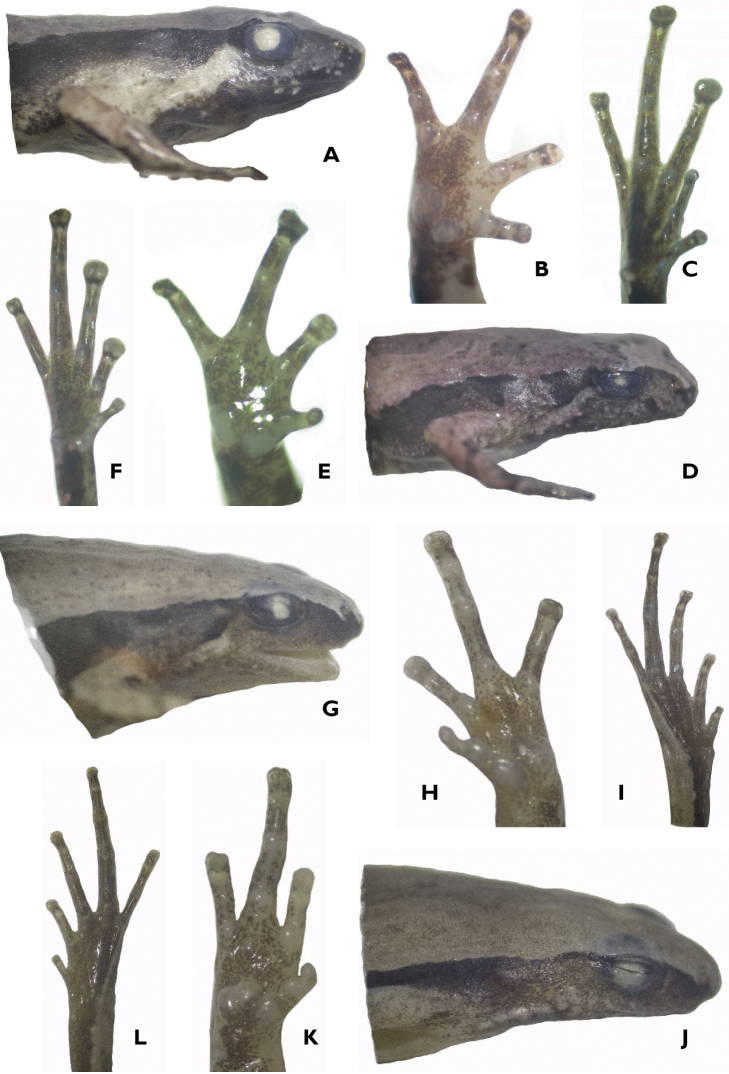
Plots of the first principal component (PC1) versus the second (PC2) for the males and the females of *Microhyla ninhthuanensis* sp. nov. (blue), *Microhyla daklakensis* sp. nov. (green), and *Microhyla ‘heymonsi*’ (red).

#### Microhyla daklakensis sp. nov.

Taxon classificationAnimaliaAnuraMicrohylidae

3A63CDD9-EE1A-5F29-B800-4A6FE88DC3C8

http://zoobank.org/F1830785-835D-45AD-8416-ABD9BBE5F27D

Figures 3K, L , 4E, F , 5G, H, I ; Table 1 ; Suppl. material 1: Tables S4, S5

##### Holotype.

VNMN 06877, adult male, collected in Nam Ka Nature Reserve, Krong No District, Dak Lak Province, Vietnam (12°20'25.39"N, 108°1'23.67"E, ca. 519 m a.s.l., Fig. 1); 27 May 2018, leg C.V. Hoang et al.

##### Paratypes.

(n = 9) All collected by C.V. Hoang et al. at the same location as the holotype: IEBR.A 4845-4746 (VNMN 06817, VNMN 06884), CIB (VNMN 06902) three adult males and VNMN 06818, CIB (VNMN 06858), ZISP 14249, 14250, 14251, 14252 (VNMN 06867, VNMN 06868, VNMN 06869, VNMN 06887), six adult females, collected on Nam Ka NR, Krong No District, Dak Lak Province, Vietnam (12°20'25.39"N, 108°1'23.67"E, ca. 519 m a.s.l., Fig. 1), 27 May 2018.

##### Diagnosis.

(1) *Microhyla daklakensis* sp. nov. is distinguished from its congeners by a combination of the following morphological characters: 1) body stocky, size medium (SVL 17.7–20.1 mm n = 4 males; 21.1–23.8 mm, n = 6 females), 2) dorsum smooth; 3) snout round in profile; 4) finger I longer than one-half the length of the finger II; 5) tips of all outer fingers dilated, forming disks, with a median longitudinal groove visible dorsally; 6) tips of all toes distinctly dilated into disks, with a weak median longitudinal groove visible dorsally, producing the appearance of two scutes; 7) inner metacarpal tubercle oval and prominent, paired outer metacarpal tubercle divided by a waistline into two equal-sized parts: outer part quite round, inner part crescent-shaped; 8) tibiotarsal articulation of straightened limb not reaching snout; 9) webbing basal: I2 – 2½II2 – 3III3 – 4IV4⅓ – 3V; 10) inner metatarsal tubercles oval, prominent and outer metatarsal tubercles round; 11) upper eyelid without supraciliary spines; 12) narrow faint brown stripe extending from rear corner of eye to axilla; 13) thin, pale vertebral stripe present, canthus rostralis with dark lines; 14) small dark round spot at mid-dorsum, divided by a light vertebral stripe; 15) dorsal surface yellowish brown, a dark brown marking in V-shape between eyes to insertion of arms; 16) vertebral and dorsolateral stripes form wavy dust strip towards the groin; 17) a small dark marking in ‘ ()’-shape on the center of the dorsum and mid-dorsal line; 18) an evenly colored black lateral stripe from above the insertion of the arms, almost reaching groin; 19) chin dark grey; throat white with scattered dark grey dusting; chest and belly creamy white.

##### Description of holotype.

Habitus stocky, size medium SVL 19.07 mm; head wider than long (HL/HW 0.82); snout long, abruptly round in dorsal view, projecting beyond margin of lower jaw, longer than diameter of eye (SL/EL 1.34); eyes comparatively small, slightly protuberant, pupil round (Fig. 5G); dorsal surface of head flat, canthus rostralis round; loreal region steep, weakly concave; nostril round, lateral, above canthus rostralis, closer to tip of snout (N-EL 1.20) than to eye (EL 1.79), interorbital distance wide (IOD 1.90) much greater than the internarial distance (IND 1.62) and the upper eyelid width (UEW 1.15); pineal spot absent, tympanum hidden, supratympanic fold weak, running from posterior corner of eye to arm insertion; vomerine teeth absent, tongue without papillae, roundly spatulate and free at the rear half of its length; slit-like openings to a median vocal sac (Fig. 6G).

Forelimbs comparatively short, about three times shorter than hindlimbs (FLL/HLL 0.33); hand two times shorter than forelimb length (HAL/FLL 0.44); fingers slender, free of webbing, round in cross-section, no skin fringes on fingers present, dorsoventrally flattened, skin fringes of fingers weak; first finger well-developed, longer than one-half the length of the second finger (1FLO/2FLO 0.57), second finger slightly shorter than fourth (2FLI/4FLI 0.89), latter much longer than first, and much shorter than third (2FLI/3FLI 0.60), relative finger lengths: I < II < IV < III (Fig. 5H); all disks bearing narrow peripheral grooves, dorsal finger tips with median longitudinal grooves producing the appearance of two scutes, grooves present in all fingers; relative finger disk widths: I < IV < II < III; nuptial pad absent; subarticular tubercles on fingers distinct, round, finger subarticular tubercle formula: 1:1:2:2 (given for fingers I:II:III:IV, respectively); inner metacarpal tubercle oval (IPTL 0.55), elongated and prominent; subarticular tubercles and outer metacarpal tubercle (OPTL 0.61) split unclearly with a weak groove (Fig. 5H).

Hindlimbs slender and slightly short (HLL 30.99), tibia length longer than half of snout-vent length (TL/SVL 0.53); tibiotarsal articulation at straightened limb not reaching snout; foot longer than tibia (FL/TL 1.36); relative toe lengths: I<II<V<III<IV; tarsus smooth, inner tarsal fold absent; tips of all toes distinctly dilated into disks, slightly wider than those of fingers (3TDW 0.63, 3FDW/3TDW 0.83), dorsally all toes with median longitudinal grooves at disks; relative toe disk widths: I<V<II<III<IV; webbing between toes basal and poorly developed (Fig. 5I), webbing formula: I2 – 2½II2 – 3III3 – 4IV4⅓ – 3V; subarticular tubercles on toes small, prominent, round, formula 1, 1, 2, 3, 2; inner metatarsal tubercle elongated, oval, large and prominent, length (IMTL 0.52) shorter than half of first toe (1TOEL 2.57); outer metatarsal tubercle round, elevated and very distinct, slightly shorter (OMTL 0.52) than length of inner metatarsal tubercle (Fig. 5I).

Skin: Dorsal surface of head and body smooth, flanks smoothly shagreened, dorsal surface of fore and hind limbs, including fingers and toes, smooth; ventral surfaces smooth (Fig. 3K, L).

##### Coloration of holotype in life.

Dorsal surface of head and trunk yellowish brown to light brown with a dark brown marking in a V-shape between eyes to insertion of arms. Vertebral and dorsolateral stripes forming a wavy dust stripe towards the groin. A small dark brown marking in ‘ ()’-shape in the center of the dorsum and mid-dorsal line. Flanks and lateral surface of head dark, a darker lateral stripe running from snout tip to nostril, fading towards the upper jaw and the belly, fading into belly as dusting. Chin dark grey; throat white with scattered dark grey dusting; chest and belly creamy white. Limbs dorsally with narrow indistinct dark brown cross-bars; fingers and toes dorsally brown with dark brown cross-bars; forelimbs ventrally creamy white, hindlimbs ventrally with creamy white thigh changing to dark grey toward shank and foot. Iris bicolored, golden in upper one-third, dark copper in lower two-thirds; pupil oval, horizontal, black (Figs 3K, 4L).

##### Coloration of holotype in preservative.

After preservation in ethanol, the dorsal coloration changed from brown to whitish grey (Fig. 4E), and the ventral surface of chest, belly, and limbs changed from creamy white to whitish beige (Fig. 4F). The dorsal pattern, dark spots on the dorsum and stripes on the dorsal surfaces of the limbs are unchanged, dark brown pattern on the dorsum changed to dark grey; iris completely black, pupil round, white.

##### Variation.

(Suppl. material 1: Table S4, Fig. 3). Paratypes vary in body size, dorsal color pattern, and shape of black scapular spots. Adult males are smaller than adult females and have a distinct vocal sac (Suppl. material 1: Table S4).

##### Comparisons.

*Microhyla daklakensis* sp. nov is morphologically similar to *M. ninhthuanensis* from Ninh Thuan Province and *M. heymonsi* sensu stricto (Fig. 2), but differs by having: 1) a snout round in profile (vs. snout obtusely pointed in *M. heymonsi* sensu stricto), 2) finger I longer than one-half the length of the finger II (vs. finger I shorter than one-half the length of the finger II in the new form from Ninh Thuan and *M. heymonsi* sensu stricto), 3) dorsal surface yellowish brown with a dark brown marking in V-shape between the eyes and the insertion of the arms (vs. pinkish brown dorsal surface with a dark brown marking in X-shape between eyes and insertion of the arms in the new form from Ninh Thuan, and dorsal surface red to grayish red with a dark brown X-shaped marking and a vague V-shaped dark brown marking in *M. heymonsi* sensu stricto (Vogt, 1913). Detailed comparisons between *Microhyla daklakensis* sp. nov. and other members of the *M. heymonsi* group are shown in Table 1 and Suppl. material 1: Table S5.

##### Etymology.

Specific epithet is in reference to the type locality, Dak Lak Province. We recommend “Dak Lak narrow-mouth frog” as the common English name and “Nhái bầu dak lak” as the Vietnamese name.

##### Natural history.

All specimens were collected at night from 19:00 to 23:00 h on the ground near the banks of small temporary ponds formed after heavy rain, along the edges of the forest and on the sides of a recently constructed road next to the devastated forests (Fig. 6A). The new species was found in sympatry with four congeners including *M. berdmorei*, *M. butleri*, *M. mukhlesuri*, and *M. pulchra*, all of which were reproducing simultaneously with the new species in the same breeding site. Other anurans such as *Fejervarya limnocharis*, Occidozyga cf. lima, and *Occidozyga martensii* also occurred in sympatry. Larval stages and eggs of the new species are unknown.

##### Distribution.

*Microhyla daklakensis* sp. nov. is currently known only from the type locality in Nam Ka Nature Reserve, Krong No District, Dak Lak Province, Vietnam (Fig. 1). The species was recorded at an elevation of ca. 500 m a.s.l.

##### Conservation status.

Currently, the evergreen forest in Nam Ka Nature Reserve, Dak Lak Province, is connected with other forests in the Tay Nguyen Plateau. The extent of its actual distribution range requires further study. Given the available information, we suggest *Microhyla daklakensis* sp. nov. be considered as Data Deficient following IUCN’s Red List categories (IUCN Standards and Petitions Subcommittee 2017).

## Discussion

Our matrilineal genealogy is consistent with those of Matsui et al. (2011), Peloso et al. (2016), Nguyen et al. (2019), Garg et al. (2019), and Gorin et al. (2020, 2021). The BI and ML genealogy showed that the monophyly of Microhylinae was not supported and the relationships among microhylid subfamilies remained unresolved (Nguyen et al. 2019) (Fig. 2). In our phylogenetic analyses using a 12S rRNA–16S rRNA gene fragment, *M. ninhthuanensis* was recovered as sister to *M. heymonsi* sensu stricto with high nodal support values (1.00/99.2) and in turn, this clade was sister to *M. daklakensis* with nodal support values (1.00/54.1). Furthermore, the results of morphometric analyses (PCA) indicated *M. ninhthuanensis* to be distinct from *M. daklakensis* and *M.* ‘*heymonsi*’.

The discovery of *M. ninhthuanensis* and *M. daklakensis* brings the total number of known species in the genus *Microhyla* to 46 and the species number in Vietnam to 12. The Truong Son Range harbors the highest diversity of the genus *Microhyla* with ten recorded species so far. It shows that there is still an underestimation of species diversity in the *Microhyla* genus (especially *M. heymonsi* group). We strongly recommend focused research to elucidate the taxonomic issues of the *M. heymonsi* group.

In terms of conservation concern, habitat loss is one of the greatest threats to amphibians in Southeast Asia, and the amphibians of the region appear to be particularly vulnerable to habitat alterations (Rowley et al. 2010a, 2010b, 2016). The need for further biological exploration in this region in concert with improved conservation measures is urgent due to intensified logging and road construction, along with increasing agricultural pressure and other human activities (De Koninck 1999; Kuznetsov and Kuznetsova 2011; Laurance 2007; Meijer 1973; Meyfroidt and Lambin 2008).

## Supplementary Material

XML Treatment for Microhyla ninhthuanensis

XML Treatment for Microhyla daklakensis

## Supplementary materials

Supplementary material 1 Tables S1–S6 Data type species data Explanation note **Table S1.** Specimens of *Microhyla* and *Nanohyla* used in the morphological and molecular analyses. **Table S2.** Specimens and sequences of *Microhyla* and outgroup Microhylidae representatives used in molecular analyses. **Table S3.** Uncorrected (“p”) distance matrix showing percentage pair-wise genetic divergence 12S rRNA16S rRNA between members of the *Microhyla* species group. **Table S4.** Measurements (in mm) and proportions of the type series of *M. neglecta*, *M. pineticola*, *Microhyla ninhthuanensis* sp. nov., *Microhyla daklakensis* sp. nov., and *M.* '*heymonsi*'. **Table S5.** Selected diagnostic characters for the species in the genus *Microhyla* (modified from Poyakov et al. 2014; Nguyen et al. 2019). All measurements are given in millimeters (mm). **Table S6.** Variable loadings for principal components with Eigenvalue greater than 0.01, from morphometric characters corrected by SVL. All measurements are given in millimeters (mm). https://binary.pensoft.net/file/539482This dataset is made available under the Open Database License (http://opendatacommons.org/licenses/odbl/1.0/). The Open Database License (ODbL) is a license agreement intended to allow users to freely share, modify, and use this Dataset while maintaining this same freedom for others, provided that the original source and author(s) are credited.Chung Van Hoang, Tao Thien Nguyen, Hoa Thi Ninh, Anh Mai Luong, Cuong The Pham, Truong Quang Nguyen, Nikolai L. Orlov, Youhua Chen, Bin Wang, Thomas Ziegler, Jianping Jiang
